# Second Generation of Antiepileptic Drugs and Oxidative Stress

**DOI:** 10.3390/ijms24043873

**Published:** 2023-02-15

**Authors:** Kamil Kośmider, Maciej Kamieniak, Stanisław J. Czuczwar, Barbara Miziak

**Affiliations:** Department of Pathophysiology, Medical University of Lublin, 20-090 Lublin, Poland

**Keywords:** epilepsy, oxidative stress, neurodegeneration, epileptogenesis

## Abstract

Epilepsy is a chronic disease of the central nervous system characterized by recurrent epileptic seizures. As a result of epileptic seizure or status epilepticus oxidants are excessively formed, which may be one of the causes of neuronal death. Given the role of oxidative stress in epileptogenesis, as well as the participation of this process in other neurological conditions, we decided to review the latest state of knowledge regarding the relationship between selected newer antiepileptic drugs (AEDs), also known as antiseizure drugs, and oxidative stress. The literature review indicates that drugs enhancing GABA-ergic transmission (e.g., vigabatrin, tiagabine, gabapentin, topiramate) or other antiepileptics (e.g., lamotrigine, levetiracetam) reduce neuronal oxidation markers. In particular, levetiracetam may produce ambiguous effects in this regard. However, when a GABA-enhancing drug was applied to the healthy tissue, it tended to increase oxidative stress markers in a dose-dependent manner. Studies on diazepam have shown that it exerts a neuroprotective effect in a “U-shaped” dose-dependent manner after excitotoxic or oxidative stress. Its lower concentrations are insufficient to protect against neuronal damage, while higher concentrations produce neurodegeneration. Therefore, a conclusion follows that newer AEDs, enhancing GABA-ergic neurotransmission, may act similarly to diazepam, causing neurodegeneration and oxidative stress when used in high doses.

## 1. Introduction

Epilepsy is a chronic disease of the central nervous system (CNS) characterized by recurrent epileptic seizures [1]. There are approximately 70 million patients suffering from epilepsy worldwide [2]. The oldest known description of an epileptic seizure comes from ancient Mesopotamia and dates back to 2000 B.C. For most of its history, epilepsy has been considered a sign of possession by evil spirits or a punishment for sins [3]. This myth may persist up to the present day, making patients feel alienated and stigmatized [4]. The International League Against Epilepsy (ILAE) divides causes of epilepsy into the following categories: structural (presence of a structural lesion that may be associated with epilepsy), genetic (there is a known mutation leading to the disease), infectious, metabolic, immune and unknown causes [5]. Epileptic seizure is the result of excessive abnormal synchronous neuronal activity in the brain, which is a consequence of the imbalance between excitation and inhibition in the CNS in favor of the former [6]. According to the classification developed by ILAE in 2017, epileptic seizures can be divided into three main categories: focal onset seizures (originating in one hemisphere), generalized onset seizures (beginning simultaneously in both hemispheres) and seizures of unknown onset [5].

Epilepsy poses a serious clinical problem and about 30% of epilepsy cases are drug-resistant [7]. This is because classical antiepileptic drugs (AEDs, also known as antiseizure drugs: phenobarbital, primidone, phenytoin, carbamazepine, and valproate) do not always provide optimal seizure control. Therefore, as a result of intensive search, new generations of AEDs have been developed. Unfortunately, the newer AEDs have not been found to be more efficient in controlling epilepsy than classical AEDs. Nevertheless, their development was a major step forward in treating epilepsy because they generally have better tolerability and less severe adverse effects. Furthermore, the majority of newer AEDs are characterized by minimal or no hepatic metabolism and thus they are less likely to interact with hepatic enzymes, unlike older AEDs [8]. Newer drugs present comparable mechanisms of action to the classical ones, and the most important among them are the increasing of inhibitory γ-aminobutyric acid GABA-ergic transmission, the inhibition of excitatory transmission (especially glutamatergic) and the modulation of voltage-gated ionic channels [8]. Some of them also affect additional brain targets (see below).

Oxidative stress is a state of homeostatic imbalance between oxidants and antioxidant factors. Typical oxidants are reactive oxygen species (ROS) and reactive nitrogen species (RNS), which are formed as by-products of various reactions in the organism. ROS and RNS are crucial for many signaling pathways, hence their proper level must be maintained. Excess of ROS/RNS may be evident when the effect of the damaging factor (e.g., inflammation) exceeds the cell’s compensation capacity. Oxidative stress is involved in the pathogenesis of many diseases, e.g., neurodegenerative diseases such as Alzheimer’s disease (AD), Parkinson’s disease (PD), Huntington’s disease (HD) and amyotrophic lateral sclerosis (ALS) [9]. Concentrations of endogenous antioxidants such as catalase (CAT), superoxide dismutase (SOD), glutathione (GSH) and glutathione peroxidase (GPx) are used to determine the intensity of oxidative stress [9].

Epileptogenesis is a continuous process leading to increased tissue susceptibility to generate spontaneous recurrent seizures, resulting in the development of epilepsy and/or the progression of previously established epilepsy. Epileptogenesis covers all biological factors causing or supporting the progression of epilepsy, such as molecular, anatomical or circuit level pathologies [10]. As a result of epileptic seizure ROS and RNS are excessively formed in the region of abnormal neuronal activity, which may be one of the causes of subsequent neuronal death. In addition, oxidative stress seems to play an important role in epileptogenesis. ROS and RNS contribute to the development of neuroinflammation, neurodegeneration and invalid neurogenesis, all three being the key mechanism causing epileptogenesis. The exposure of neurons to oxidative stress may cause their increased susceptibility to seizure generation, whilst the administration of antioxidants may be a preventive procedure [9].

Given the role of oxidative stress in epileptogenesis, as well as the participation of this process in other neurological diseases and conditions, we decided to review the latest state of knowledge regarding the relationship between selected newer AEDs (vigabatrin (VGB), levetiracetam (LEV), gabapentin (GBP), tiagabine (TGB), lamotrigine (LTG), oxcarbazepine (OXC), topiramate (TPM), felbamate (PBM)), and the generation of free radicals.

In order to collect all the necessary data, we conducted a conscientious search in the US National Library of Medicine (PubMed) database. Although we preferred the most recent papers possible (not more than five years old at the time of writing), we occasionally used older studies in the face of insufficient data on particular topics. In order to find the most relevant papers, the search terms were respective drug names AND “oxidative stress”.

## 2. Newer Antiepileptic Drugs

### 2.1. Vigabatrin (VGB)

VGB was approved for clinical use in the United Kingdom in 1989. As an analogue of GABA, it irreversibly inhibits the action of GABA-transaminase (GABA-T), thus increasing the concentration of this inhibitory neurotransmitter in the brain. It is used for the management of infantile spasms (IS) and refractory complex partial seizures (CPS) [11]. The most common adverse effects are sedation and fatigue, occurring in 13% and 8% of patients, respectively. Other adverse effects include dizziness, confusion, memory impairment, headache, ataxia, or diplopia [12].

In 2005, studies were conducted to check the effect of VGB on the oxidation of proteins and lipids, as well as on GSH, GPx, and glutathione-transferase (GST) levels in the liver of rat fetuses born to mothers receiving 100 mg/kg/day of VGB at various stages of pregnancy. Fertilized rats were divided into four groups; group I received VGB in the first week of pregnancy, group II in the second, group III in the third, and group IV served as a control. Rats were sacrificed and samples were taken on the 22nd day of gestation. After examining the fetal liver samples, it turned out that the protein oxidation in groups I and II was significantly higher than in the control group. In addition, in groups I, II and III the level of lipid peroxidation and the activity of GPx was also higher than in group IV. The level of GSH in groups I and II was significantly lower than in the control group, while GST was only increased in group I and no significant differences were found in groups II and III. Group III also did not differ significantly from the control group in protein oxidation and GSH level. These studies clearly indicate that VGB passes through the placenta and causes oxidative stress in the rat’s fetal liver. This is particularly noticeable in the first week of pregnancy (which corresponds to the first trimester in humans), but it is also important in the second and third week of the rat pregnancy. These results also reveal that defense mechanisms that neutralize ROS/RNS are insufficient. This points to the possible teratogenic effect of VGB. Therefore, the authors of these studies do not recommend using this AED in the first trimester of pregnancy in women with epilepsy [13].

It has been proven that excessive concentration of GABA causes the inhibition of mitophagy and pexophagy, which results in an enhancement in the number of mitochondria and peroxisomes [14]. The administration of VGB results in an increase in the level of GABA in the eye and in the brain, and this increase is dose-dependent. In addition, the administration of VGB not only produced an enhancement in the number of mitochondria, but also an increase in their size. Studies of total GSH and malondialdehyde (MDA, lipid peroxidation marker) showed an increase in the oxidative stress levels in the eye following VGB. Therefore, these studies have confirmed the link between VGB administration and mitochondrial dysfunction. The reported changes are suspected as the cause of visual field defects in patients receiving VGB [15].

Preclinical animal data prove that ganaxolone, a positive allosteric modulator of GABA-A receptor complex, and acamprosate, a GABA agonist acting by both GABA-A and GABA-B receptors, exhibit therapeutic potential in FXS [16,17]. A phase II randomized controlled clinical trial of ganaxolone conducted on 59 children aged 6–17 revealed that this drug had a positive effect on anxiety, attention, and hyperactivity in children with a higher level of anxiety and lower cognitive function [18]. Whether this positive therapeutic effect could be better when both GABA-A and GABA-B receptors are modulated remains to be determined. Several studies have reported that VGB exerts a neuroprotective effect during neuronal ischemia [19,20,21]. Neuroprotection may occur by increasing GABA-ergic transmission because GABA causes a decrease in glutamatergic transmission via the activation of GABA-A and GABA-B receptors [19,20,21,22]. Glutamate excitotoxicity is a major factor in ischemic conditions and is responsible for neuronal death [23]. A study has been conducted to test whether VGB’s neuroprotection may be used to mitigate the consequences of spinal cord ischemia-reperfusion injury [24]. New Zealand rabbits (N = 24) were divided into four groups. The first group served as a control, and rabbits from other groups underwent cross-clamping on the abdominal aorta in order to induce spinal ischemia. One group after surgery received 50 mg/kg of VGB (low-dose group, similar to optimum clinical use), another 150 mg/kg (high-dose group, maximum dose in the clinical use), and the last group received 25 mL of physiological fluid (ischemia-reperfusion group). After 48 h of neurological control (motor assessment using a Tarlov numerical scale) all rabbits were sacrificed. Levels of MDA, advanced oxidation protein products (AOPP, marker of protein oxidation), nitric oxide (NO) and GSH and SOD activities in plasma and tissue samples were analyzed, and a histopathologic assessment of ischemic tissues was also performed [24]. In the ischemia-reperfusion group, all parameters of oxidative stress (MDA, AOPP, NO) were significantly increased compared to the control group, both in tissues and in plasma. Furthermore, there was a decrease in SOD and GSH activities. VGB therapy, irrespective of the dose, caused a significant decrease in the level of MDA, AOPP and NO, almost to the control group values. There was also a significant increase in SOD and GSH activities following VGB administration, both in tissues and plasma. VGB neuroprotection was confirmed by histopathological examination, which showed a reduced number of damaged neurons, axons and a lower level of astrocyte-microglia infiltration in the low-dose group. Similar results were obtained in the high-dose group, with a difference in axonal damage; in this group, severe vacuolization of axons occurred, probably due to VGB toxicity. It is worth noting that the motor function assessment using the Tarlov scale demonstrated that the low-dose group had slightly better performance at 24 and 48 h assessments compared to the ischemia-reperfusion group; nonetheless, these results were not statistically significant (*p* = 0.396 and 0.214, respectively). This study found the antioxidant potential of VGB and the ability of this drug to alleviate the consequences of spinal cord ischemia [24]. These effects can be especially helpful in the treatment of secondary injury in the course of spinal cord injury, where glutamate excitotoxicity and oxidative stress are important pathophysiological factors [25]. However, further studies are needed to assess the clinical potential of VGB, especially given the fact that significant histopathological and biochemical improvement did not correspond with the statistically significant neurological recovery [24]. It should be noted that other AEDs, by increasing GABA-ergic transmission or by reducing glutamatergic activity, may also exert beneficial effects in the treatment of ischemic neuronal damage [22].

### 2.2. Lamotrigine (LTG)

LTG [6-(2,3-Dichlorophenyl)-1,2,4-triazine-3,5-diamine] is a multifunctional phenyltriazine derivative. It works by inhibiting voltage-gated sodium channels, which in turn reduces the secretion of excitatory amino acids and primarily glutamate. This AED also stabilizes neuronal membranes [26]. Moreover, LTG has been documented to effectively block α-amino-3-hydroxy-5-methyl-4-isoxazolpropionic acid (AMPA) glutamate receptors [27]. LTG may be used in partial seizures with or without secondarily generalized tonic-clonic seizures, as well as in Lennox–Gastaut syndrome [27]. There are differences in the tolerance of immediate-release (IR) and extended-release (XR) LTG in the favor of the latter. The most common adverse effects are dizziness (IR: 35% of patients, XR: 19%), diplopia (IR: 25%, XR: 4%), ataxia (IR: 20%, XR: 5%) and nausea (IR: 19%, XR: 7%), and others include blurred vision, somnolence, rash and vomiting. A relatively rare complication of treatment with this drug may be Stevens–Johnson syndrome, occurring in 0.1% of patients taking IR LTG [28].

The effect of LTG on oxidative stress has been the subject of many studies. Stress intensity could be assessed through MDA level and/or reduced concentrations of GSH, CAT and SOD. The effects of LTG and carbamazepine were evaluated on cognitive functions in rats and oxidative stress in the rat brain during chemically induced seizures. Convulsions were kindled by the administration of pentylenetetrazole (PTZ). The study revealed that LTG administration in the group with PTZ-kindled epileptogenesis significantly decreased MDA level, and also increased GSH, SOD and CAT activities in the homogenized whole brain samples as compared to the carbamazepine-treated group [29].

The concentrations of MDA and GSH were significantly higher in the epileptic rats in the model of PTZ-induced kindling compared with the control group. The administration of LTG reduced the level of these indicators, and in addition the AED protected against the hippocampal damage [30].

A recently conducted study compared the blood level of reactive oxygen metabolites (d-ROMs) and biological antioxidant potential (BAP) in patients treated with conventional AEDs and those who additionally received LTG and LEV. Older AEDs caused a significant increase in d-ROMs levels, in contrast to the results obtained in the group treated with older AEDs combined with LTG and LEV, where a decrease in d-ROMs levels and an increase in BAP were achieved [31].

### 2.3. Tiagabine (TGB)

TGB is a newer AED that was authorized for use in many European countries in the 1990s. It selectively inhibits glial and neuronal GABA-uptake in the CNS by blocking a GABA transporter GAT 1. The blockage of GAT 1 causes an increase in intra-synaptic concentrations of GABA, which leads to an enhancement in the number of inhibitory signals in neurons [27]. It is used in patients over 12 years of age for the treatment of focal and secondarily generalized seizures. The most common side effects of TGB are CNS-related, including dizziness (in 30% of patients), asthenia (24%), nervousness (12%), tremor (9%), depression (5%) and emotional lability (4%). However, no loss of visual field has been observed, which occurs in some patients using VGB [32].

The use of TGB in the treatment of HD has been investigated. In the animal model of HD, disorders typical for this disease are caused by an inhibitor of the mitochondrial citric acid cycle: 3-nitropropionic acid (3-NP) [33]. Oxidative stress occurred in rats treated with 3-NP, as evidenced by elevated MDA, increased NO levels, and a reduced amount of GSH in the whole brain samples compared to the control group. The administration of TGB prior to the administration of 3-NP weakened the increase in the level of oxidative stress indicators [34].

In 2000, the results of research on the influence of TGB on primary rat cortical astrocyte cultures were published [35]. The study comprised five groups: one control, two with TGB being added at 1 μg/mL or 10 μg/mL, and two in which TGB was dissolved with lipopolysaccharides (LPS) for the induction of oxidative stress. It turned out that, in the group where TGB (1 μg/mL and 10 μg/mL) was co-administered with LPS, there was a lower level of oxidants than in the group where LPS was administered alone [35].

On the other hand, the same study has shown that a higher concentration of TGB (10 μg/mL) may result in an increased production of ROS (but not NO) in cells. This effect did not occur at therapeutic concentrations of TGB or when TGB was co-administered with LPS [35]. In another study, TGB’s toxic effect on the DNA of cortical rat astrocytes culture was evaluated. TGB at 1 and 10 μg/mL did not cause DNA damage, however, when cells were exposed to 20 μg/mL and 50 μg/mL; DNA damage has been detected in a dose-dependent way [36].

### 2.4. Gabapentin (GBP)

GBP, 1-(aminomethyl)-cyclohexaneacetic acid, is an AED binding to the α2δ subunit of voltage-gated Ca^2+^ channels (VGCC), leading to VGCC dysfunction and the suppression of depolarization-induced Ca^2+^ influx. The inhibitory effect of this drug on seizure activity occurs mainly by blocking presynaptic P/Q-type Ca^2+^ channels, which predominate in glutamatergic terminals. This leads to a reduction in glutamate release and, consequently, decreased neuronal excitability [37]. Moreover, GBP has been proven to cause elevation of intracellular GABA concentration in the occipital lobe of the human brain. This effect is most evident after the first dose of GBP and decreases during chronic GBP therapy; however, it remains significantly higher than the initial GABA concentration [38]. GBP can elevate GABA synthesis in the brain by increasing glutamate decarboxylase activity. Furthermore, GBP reduces the activity of branched-chain aminotransferase (BCAT), thus reducing glutamate synthesis [37]. GBP is recommended against partial epilepsy with/without secondary generalization and can be used in patients over three years of age. In addition, it may be used for neuropathic pain, restless legs syndrome, fibromyalgia, trigeminal neuralgia, multiple sclerosis, headache, anxiety, and post-operative pain [39]. In general, GBP is well tolerated and its few adverse effects include somnolence (in 19% of patients), dizziness (17%), ataxia (13%), fatigue (11%), nystagmus (8%) and headache (8%). In addition, GBP can cause addiction, especially in people with history of excessive drinking or other stimulant use [40].

The neuroprotective effects of GBP in streptozotocine-induced diabetic rats have been investigated. Brain injury in rats was assessed by measuring the level of S100B protein (present mainly in astrocytes) and neuron-specific enolase protein (NSE, located primarily in neurons), which are reliable markers of brain damage. The level of glial fibrillary acidic protein (GFAP) was also examined, which, like S100B, is an astrocytic marker and is crucial in a process called reactive gliosis that occurs in response to brain damage. The level of oxidative stress was also evaluated by measuring the level of lipid peroxidation and GSH. The results indicate that GBP exerts neuroprotective effects. The administration of GBP at 50 mg/kg/day for six weeks prevented the growth of both glial and neuronal markers that occur in the course of diabetes. In addition, a reduction in oxidative stress markers has been achieved. Looking for the causes of the neuroprotective action of GBP, the hypothesis was assumed that it was due to a decrease in the oxidative stress level [41]. Oxidative stress, as shown by previous studies on streptozotocin-induced diabetic rats, is one of the most important causes of gliosis and neuronal degeneration in diabetes [42]. Hence, the antioxidant effect of GBP can be linked to the normalization of elevated glial and neuronal markers.

The above effect exerted by GBP probably results from two mechanisms. The first one involves increasing the concentration of GABA in the brain, which is reduced as a result of hyperglycemia [43]. Increased GABA concentration is associated with enhanced inhibitory transmission, resulting in neuroprotective effects [41]. The second mechanism includes a reduction in glutamatergic transmission [44]. An increase in glutamatergic secretion and glutamate receptor (*N*-methyl-d-aspartate—NMDA) activation was observed in the course of diabetes [45]. It has been confirmed that enhanced glutamatergic transmission induces oxidative stress, while free radicals damage neurons and glial cells, causing reactive gliosis and the production of GFAP and S100B by glial cells [46]. GBP prevents these pathologies by inhibiting glutamatergic transmission, and thus reduces oxidative stress [41].

Excessive glutamate in synaptic clefts and its excitotoxicity are associated with the development of oxidative stress that causes apoptosis [47]. GBP is a leucine analogue, as well as a specific inhibitor of BCATc; thus, it prevents the formation of excessive BCAAs in retinal neurons. Given the negative effects of excessive glutamate in the course of diabetes, it has been hypothesized that GBP may have a neuroprotective effect on retinal cells in streptozotocin-induced diabetic rats [48]. The study demonstrated that diabetic rat retinas exhibit a significant increase in BCAAs compared to the control group of healthy rats, BCKAs levels being similar in both groups. GBP treatment (300 mg/kg/day for two weeks) of diabetic rats resulted in a significant decrease in BCAAs levels as well as a slight increase in BCKAs concentrations. Diabetic rats also exhibited a 40% reduction in GSH levels, as well as an almost two-fold increase in MDA levels compared to healthy rats. GBP therapy of diabetic rats resulted in an increase in GSH concentration almost to the control value, as well as a significant reduction in MDA levels [48]. The expressions of retinal proapoptotic caspase-3, which correlates with the activity of apoptotic processes [49], as well as Bcl-2 protein, which has anti-apoptotic properties [50], were also examined. As expected, caspase-3 expression in non-treated diabetic rats was 32% higher than in the control, whereas in diabetic rats treated with GBP caspase-3 expression was comparable to the control group. In addition, Bcl-2 expression was significantly reduced in diabetic rats compared to the control group; however, in rats treated with GBP, the expression of Bcl-2 was significantly higher than in non-treated diabetic rats [48]. The authors of the study developed a method for measuring the level of ROS in the retina under in vivo conditions. It involves injecting carboxy-H2DCFDA into the vitreous body of the rat, which after oxidation by ROS shows fluorescent properties. Fluorescence was measured after 6 h when the full diffusion of the substance into the rat retina had occurred. It was estimated that in the retina of diabetic rats ROS levels were increased by almost 55% compared to healthy retinas. GBP therapy evidently normalized ROS concentrations in diabetic retinas [48].

GBP’s influence on oxidative damage in mice with hypoxic stress was evaluated. Pretreatment with GBP (50 or 100 mg/kg, 30 min prior to hypoxic stress) reduced brain MDA and nitrite levels; it also resulted in increased GSH and CAT activity in the whole brain homogenate [51]. Furthermore, the same study revealed that GBP increased locomotor activity and reduced anxiety in the stressed mouse. The authors of this study also indicate that the neuroprotective properties of GBP result from elevated GABA-mediated inhibition and reduced glutamatergic neurotransmission [51].

Similar conclusions were reached by examining the impact of GBP on behavioral alterations and oxidative damage in mice with immobilization stress. In this case, neuroprotective effects of GBP (pretreatment with 50 mg/kg or 100 mg/kg) have also been found, manifested by lowering oxidative damage (reduced MDA and nitrite levels, increased GSH level and GSH activity in the brain homogenate) or preventing behavioral alterations. Furthermore, pretreatment with picrotoxin (a GABA-A receptor antagonist) resulted in the abolition of the neuroprotective effect while pretreatment with muscimol (a GABA-A agonist) resulted in a further decrease in MDA and nitrite concentrations and the elevation of reduced GSH levels and CAT activity [52].

The effect of GBP and LTG on abnormalities resulting from the administration of the 3-NP to rats (an animal model of HD) was investigated. Treatment with GBP (50 and 100 mg/kg) or LTG (10, 20 and 40 mg/kg) resulted in a decrease in striatal MDA, nitrite concentration, and restored SOD and CAT activities compared to animals which received 3-NP alone [53].

The evaluation of some AEDs’ effects on oxidative stress has shown that GBP diminished elevated NO levels generated by PTZ-induced convulsions, this effect being particularly noticeable in the hippocampus and cortex [54]. Similar results were obtained in a study conducted to estimate the efficiency of hesperidin (Hesp), a bioflavonoid with proven antioxidant and anti-inflammatory properties, in mitigating PTZ-induced convulsions in mice. After the intraperitoneal administration of PTZ (80 mg/kg) and behavioral evaluation, the animals were sacrificed. PTZ injection in the control group resulted in seizures, increased MDA and nitrite levels, reduced SOD, GSH and CAT activities, and activities of mitochondrial complex enzymes (I, II and IV) in the brain tissue homogenates. Pretreatment with Hesp (at the dose of 200 mg/kg/day per os) for one week prior to the PTZ administration delayed latency for the onset of convulsions, diminished nitrite and MDA levels and increased activities of antioxidant and mitochondrial enzymes, as compared to the PTZ group. Similar results were also obtained after a single intraperitoneal injection of GBP at the dose of 20 mg/kg or 0.5 mg/kg of diazepam 30 min before PTZ. Diazepam and GBP caused a significant reduction in oxidative stress markers, raised SOD, GSH and CAT activities, and increased seizure threshold in comparison with the control group. The administration of GBP and diazepam was also found to restore activities of mitochondrial complex enzymes. The positive action was not observed when GBP, diazepam or Hesp were used at lower doses of 10 mg/kg, 0.2 mg/kg and 100 mg/kg/day, respectively. However, pretreatment using Hesp (100 mg/kg/day) combined with GBP (10 mg/kg) was found to induce a significant anticonvulsant effect as compared to the control group, similar results being obtained when Hesp (100 mg/kg/day) was co-administered with diazepam (0.2 mg/kg). Moreover, in both cotreatment groups there were significant increases in concentrations of antioxidant enzymes and the activities of mitochondrial enzymes as compared to the PTZ group. The results of this study indicate that Hesp, GBP and diazepam efficiently reduce oxidative stress in the model of PTZ-induced seizures in mice. Furthermore, when used in subtherapeutic doses, Hesp co-administered with GBP or diazepam also exerts significant antioxidative and anticonvulsant effects. Therefore, it was concluded that Hesp might potentiate antioxidant and anticonvulsant effects of GBP and diazepam [55].

GBP inhibits oxidative stress associated with neuropathic pain. Neuropathic pain was triggered in Wistar rats through partial sciatic nerve ligation. The observation of lipid peroxidation, reduced GSH level, SOD and CAT activity showed that GBP (100 mg/kg) attenuated oxidative stress. It was also found that GBP reversed hyperalgesia and cold hyperalgesia and significantly reduced damage to myelinated and unmyelinated fibers [56].

Further experimental evidence demonstrated that GBP (pretreatment with 1 mg/kg) reduced paw oedema induced by carrageenan or dextran. GBP also elevated GSH level and reduced MDA concentration in the peritoneal fluid [57]. The anti-inflammatory effect of GBP results from the inhibition of inflammatory mediators, neutrophil migration into the inflammatory site and proinflammatory cytokines. This effect is also caused by reduction of oxidative stress; GBP increased the GSH level and reduced MDA concentration [57].

In the study of the GBP effect on cerebral ischemia reperfusion injury in a middle cerebral artery occlusion rat model, GBP exerted clear cut neuroprotective properties [58]. Pretreatment with GBP (150 mg/kg) caused a significant reduction in ischemia-reperfusion-induced neuronal death and reduced oxidative stress markers in the penumbra. Western-blot analysis indicated that phosphoinositide-3-kinase (PI3K)/protein kinase B (Akt)/mammalian target of rapamycin (mTOR) signaling pathway was activated by GBP [58]. The activation of the PI3K/Akt/mTOR signal pathway has been proven to possess neuroprotective effects [59]. In vitro studies have confirmed that GBP causes activation of this signaling pathway, which reduces apoptosis and oxidative stress in the oxygen-glucose deprivation model. Moreover, the neuroprotective effect of GBP was suppressed by LY294002, an inhibitor of PI3Ks. The authors of the study concluded that the neuroprotective effect of GBP in the middle cerebral artery occlusion rat model resulted from the activation of PI3K/Akt/mTOR signaling pathway, thus describing another mechanism in which GBP caused a reduction in oxidative stress and, consequently, a reduction in neuronal apoptosis [58].

GBP and TGB are recommended as first-line co-analgesic drugs in the treatment of cancer pain, effectively reducing suffering and providing improvement in sleep and quality of life, and also allowing a reduction in opioid doses [60]. Whether GBP could affect tumor progress was investigated by assessing the effect of GBP administration on Ehrlich tumor growth in Swiss mice [61]. A decrease in SOD activity and impaired free radical removal promotes tumor progress [62]. This study demonstrated that the administration of GBP (100 mg/kg) increased SOD activity in the ascitic fluid. Moreover, a further examination of ascitic fluid revealed a reduction in arginase activity following GBP administration and, apparently, an increase in arginase activity is associated with tumor growth. The study also showed that GBP therapy caused an increase in IL-6 and MCP-1 serum levels that affect chemotaxis and macrophage activation, which may reduce tumor growth. Differences in the tumor cell count between the control and GBP treated mice were not observed. However, GBP therapy increased the weight of the mice compared to the control, and importantly, this was not caused by ascites or tumor progress. The authors are of the opinion that despite GBP therapy not affecting tumor development, the effects of GBP at the molecular level, such as increased SOD activity, IL-6 and MCP-1 levels and reduced arginase activity, may have a positive effect on the patients’ general condition [61].

Different results were obtained by investigating the effect of GBP on oxidative stress in a model of toxic demyelination in the rat brain, caused by the intracerebral injection of ethidium bromide. Rats were divided into three groups: group I received 100 mg/kg of GBP daily for 10 days prior to ethidium bromide injection, group II received 300 mg/kg of GBP daily for 10 days before ethidium bromide administration, and rats in the control group were given saline instead of GBP. The animals were sacrificed the day after the intracerebral injection of ethidium bromide. In the control group, an increase by 30.2% in MDA level, GSH decrease by 17.6 %, and the inhibition of GPx (by 78.6%) and paraoxonase activity (by 27.5%) as well as an increase by 55.4% in the brain cortex nitrite concentrations were observed. After pretreatment with GBP at 300 mg/kg, cortical MDA was increased by 66% and the GPx activity was inhibited by 54.3%. There was also a decrease in paraoxonase activity by 83.3% and a 29.2% decrease in nitrite, which is the end product of NO metabolism, pointing to a reduced level of NO compared to the control group. The GSH level was not changed after pretreatment with GBP, irrespective of the dose used. Despite the decrease in nitrite levels, other changes observed in the brain cortex show that GBP at 300 mg/kg increased lipid peroxidation and decreased the activity of antioxidant enzymes in this model of toxic demyelination. Following pretreatment with GBP at 100 mg/kg, there was a 73% decrease in paraoxonase activity and a 21.4% reduction in brain nitrite compared to the control group. However, no significant differences were found in MDA level and GPx activity [60]. Other studies have shown that GBP at high concentrations are able to cause oxidative stress in rat astrocyte cultures. For instance, GBP at a concentration of 50 μg/mL or 100 μg/mL produced a significant elevation of NO accumulation and ROS production and an increase in the level of fat peroxidation. DNA damage also occurred. However, lower GBP concentrations (1 μg/mL, 10 μg/mL) are well tolerated by cortical astrocytes [63].

On the other hand, blocking GABA transporters was not beneficial in mitigating the consequences of ischemia during preclinical studies. C57BL/6 J male mice with focal motor cortex ischemia induced by photothrombosis were divided into four groups, which were given two intraperitoneal doses (1 and 6 h post-ischemia) of the GAT 1 inhibitor, TGB (1 mg/kg or 10 mg/kg), the GAT2/3 inhibitor (S)-SNAP-5114 (5 or 30 mg/kg), the GAT1/BGT1 inhibitor EF-1502 (1 or 10 mg/kg) or vehicle (2% or 10% DMSO in saline, control group). In all groups that were given GAT inhibitors, no significant improvement in infarct volumes was assessed after seven days post-stroke, as compared to the control group. GAT inhibitors did not restore motor functions in comparison to the animals which were given the vehicle, as proven by the assessment of foot faults. There was, however, a significant decrease in forelimb asymmetry in the animals which received TGB at the dose of 1 mg. Nevertheless, TGB caused increased seizure activity post-stroke, and thus its clinical use may be limited in this regard [64]. A study was conducted to examine the effectiveness of AEDs in the treatment of seizures occurring after exposure to hyperbaric oxygen (HBO2). Such disorders may occur in divers or in patients treated with hyperbaric oxygen therapy. In this model, seizures were due to the toxic effects of HBO2 [65]. It has been shown that, after exposure to HBO2, the level of ROS/RNS in CA1 hippocampal slices is increased [66]. ROS and RNS attack redox-sensitive neuronal targets, which include ion-sensitive channels and voltage-sensitive channels for Na^+^, K^+^, Ca^2+^ ions and elements involved in the production and metabolism of neurotransmitters [65]. Damage caused by ROS and RNS eventually results in the lack of a proper ratio of inhibitory and excitatory signals, which leads to convulsions [67]. The use of VGB and TGB, which results in the increasing the concentration of GABA in the striatum, produced a more than threefold extension of the seizure latency period. A similar effect was obtained by the use of Na^+^ channel inhibitors: carbamazepine and LTG [65].

### 2.5. Oxcarbazepine (OXC)

OXC is a carboxamide derivative, a carbamazepine ketone analog which was patented in 1969 by J. R. Geigy Ltd. It is administered orally and completely absorbed in the gastrointestinal tract. It undergoes rapid conversion to its active form, i.e., 10-monohydroxy derivative, which is then eliminated by the kidneys [65]. Its mechanism of action is similar to that of carbamazepine and comprises blocking voltage-dependent sodium channels and stabilizing membranes [27]. However, unlike carbamazepine, OXC activates cytochrome P450 enzymes to a lesser extent [68]. It can be used for the management of partial seizures [69]. The most common side effects are somnolence, fatigue, sedation, dizziness and rash, each one occurring in about 6% of patients [69].

The effect of OXC on oxidative stress is quite significant. GPx, CAT, SOD and MDA were used as indicators of the effect of this AED on antioxidant mechanisms and were compared in the group of patients with epilepsy and in the control group before and after the annual treatment with OXC. However, the blood level of GPx and SOD was slightly elevated and there was no significant difference in the levels of other enzymes [70]. In addition, other studies have shown the reduction of, among others, GSH level in the brain homogenate of albino Swiss strain mice in PTZ-kindled epilepsy, despite OXC administration [71].

### 2.6. Topiramate (TPM)

TPM was discovered in 1979 and is a monosaccharide derivative. Administered orally, it is rapidly absorbed and mostly excreted unchanged by the kidneys. Its mechanism of action is based on several aspects. First, it increases the activation of GABA-A receptors by GABA. Secondly, it blocks AMPA glutamatergic receptors. It also reduces voltage-dependent sodium currents [27]. TPM can be used in many types of epileptic seizures, including in the management of drug resistant epilepsy. Common adverse effects include dizziness (occurring in 31% of patients), somnolence (28%), headaches (27%), and psychomotor impairment (20%). It is worth noting that speech disturbances and language impairment occurring in 13% and 10% of patients, respectively, pose a significant challenge in clinical practice [40].

In mice, studies were carried out to measure the level of GSH and 4-hydroxy-2-trans-noneal (HNE: a product of lipid peroxidation) in the homogenized brain of diabetic mice, thus evaluating the effect of the drug on oxidative stress caused by hyperglycemia. As was shown, TPM (at 1.0 mg/kg) lowered HNE, increased the level of GSH (from 10.8 ± 0.4 to 12.6 ± 0.6 nmol/mg protein) and improved learning behavior, thus efficiently protecting the brain from the diabetic damage [72]. However, the above-mentioned study on PTZ-induced convulsions in mice revealed that when GSH concentration was significantly reduced by TPM pretreatment, MDA level was increased, which suggested negative oxidative stress modulation by TPM [71]. However, two years later another study presented TPM-induced increased levels of GPx and GSH levels in the hippocampal tissue, concluding that the neuroprotective effects of this drug, such as the prevention of cocaine-induced memory and learning impairment in rats, seemed to be associated with its direct antioxidant activity [73,74].

### 2.7. Felbamate (FBM)

FBM is a dicarbamate with an unusual mode of action. It is an NMDA receptor antagonist, which blocks its activation by glycine. It was also documented to enhance GABA-mediated events in hippocampal neurons [27]. Unfortunately, its use is quite limited by the observed adverse effects. One of the most serious, although not frequent, is severe aplastic anemia [75]. Therefore, the use of this AED is mainly limited to patients who are resistant to other antiepileptics and as an adjuvant in Lennox–Gastaut syndrome [76].

There are very few reports regarding the effect of this drug on oxidative stress. One of them demonstrated the antioxidative potential of FBM in the PTZ-induced kindling mouse model. Treatment with FBM or LEV not only reduced convulsions but also diminished oxidative stress, as proven by a decrease in NO concentration, NO synthase activity, peroxide levels and MDA concentration in the homogenized brain tissue [77].

### 2.8. Levetiracetam (LEV)

LEV [(S)-2-(2-oxopyrrolidin-1-yl) butanamide] is quite a unique drug when it comes to the treatment of epilepsy, as well as a relatively new one, known since 2000. Its mechanism has not been fully explained: it binds to synaptic vesicle glycoprotein 2A (SV2A) [78], which plays a pivotal role in the formation of presynaptic calcium currents necessary to release neurotransmitters in the Ca^2+^-induced exocytosis process [79]. In this mechanism, it inhibits calcium ionic currents by reducing the release of neurotransmitters and thereby diminishing neuronal excitability [80]. It has been observed that LEV inhibits both GABA-ergic and glutamatergic currents in the hippocampal slices of the rat brain. This effect was the most noticeable in rapid-discharging neurons [81]. On the other hand, LEV restores the GABA/glutamate balance in the pilocarpine model of focal limbic seizures in rats. LEV causes significant elevation of GABA vesicular release in the dorsal hippocampus after SE, thus exerting an effect opposite to its action in healthy tissue. This may be associated with the participation of SV2A in GABA release in epileptic tissue [82]. Missense mutation of the SV2A gene was proven to cause increased seizure susceptibility related to impairment in GABA (but not glutamate) release in the amygdala after PTZ-induced seizures [83]. Furthermore, LEV inhibits the AMPA receptors in mouse cortical neuronal cultures, resulting in a decrease in excitatory postsynaptic currents. LEV alleviated kainate-induced currents, as well as the excitatory neurotransmission caused by an AMPA agonist [84]. Despite the increasing knowledge about the effects of LEV, the exact mechanism of its action has still not been fully explained. LEV can be used either orally or in the form of intravenous injection. It is quickly and completely absorbed in the intestines, along with being characterized by the lower chance of pharmacokinetic interactions with other drugs, due to the absence of hepatic metabolism as well as low binding with seral proteins (less than 10%). Indeed, LEV was shown not to interact with phenytoin, warfarin, digoxin and oral contraceptives [85]. It has a small spectrum of side effects, being an extremely safe drug [86]. The most common adverse effects, occurring in >10% of patients, include drowsiness, fatigue and headache [9,87]. In clinical practice, it is mainly used for the treatment of drug-resistant partial seizures [27].

The positive effect of LEV and FBM on oxidative stress was mentioned in the previous section related to studies in mice in a PTZ-induced kindling model [77]. However, other data indicate that during treatment with this drug a reduced level of GSH and increased levels of MDA are observed in the rat brain (frontal cortex and striatum) homogenate, and this AED generally increases oxidative stress [88].

In a study conducted in Turkey among children with epilepsy, the parameters of oxidative stress during LEV treatment were assessed. The LEV group consisted of 26 patients on therapeutic doses of LEV and was compared to the control group of 26 healthy volunteers. In the LEV group, elevations of MDA and 8-OHdG (8-hydroxy-2-dexyguanoisine; one of the major products of DNA oxidation) venous blood levels were observed, which points to oxidative damage [89].

## 3. Conclusions

The results presented above clearly demonstrate that VGB induces oxidative stress under normal conditions [13,15]. This is evident when VGB is administered to rat fetuses, due to their poorly developed mechanisms of protection against oxidative stress [13]. On the other hand, in the case of increased glutamatergic transmission, which occurs in Fragile X syndrome or spinal cord injury, VGB reduces oxidative stress by increasing GABA-ergic transmission [24]. In these cases, it is best to use moderate doses of VGB due to the high dose (150 mg/kg) toxicity [24]. Clinical studies on the arbaclofen and ganaxolone indicate that GABA agonists have potential for treating FXS, and thus AEDs increasing GABA-ergic transmission, such as VGB, TGB, GBP, TPM, may also be beneficial [16,17,18]. Therefore, research on the potential of newer AEDs in the management of FXS is highly desirable, especially in the pediatric population.

The available evidence points to the antioxidative effect of TGB [35,36]. However, the administration of TGB at high concentrations produces opposite results in the healthy rat primary cortical astrocyte cultures. In fact, ROS production increases and oxidative stress occurs, which causes damage to cellular structures, e.g., DNA [35,36].

GBP effectively prevents oxidative stress in various animal models [41,48,51,52,53,54,55,56,57,58,61]. Oxidative stress is associated with gliosis and neuronal damage that leads to apoptosis [90]. GBP neuroprotection probably results from increasing GABA-ergic transmission and reducing glutamate excitotoxicity [41,48,51,52,53]. GBP can activate the PI3K/Akt/mTOR signaling pathway, which may explain the reduction of oxidative stress after GBP administration in the spinal cord ischemia-reperfusion injury model [58]. Moreover, GBP at the dose of 300 mg/kg, via the inhibition of BCATc, causes the reduction of oxidative stress in a state of disturbed metabolism of glutamate in the course of diabetes, thus presenting another mechanism of neuroprotection in diabetes [48]. Additionally, GBP restores the activities of mitochondrial complex enzymes after PTZ injection [55]. This mitochondrial action is particularly important, given the fact that the impairment of the mitochondrial electron transport chain caused by ROS leads to further overproduction of ROS, thus creating a vicious circle enhancing oxidative damage. Hence, restoring mitochondrial homeostasis seems to be an encouraging strategy in protecting against seizure-induced oxidative stress [91]. It should be emphasized that, in most studies confirming the antioxidative activity of GBP, this drug was administered in doses up to 100 mg/kg [41,51,52,53,54,55,56,57,58,61].

There are reports available on the opposite effect of GBP, which, especially in high doses, may induce oxidative stress [60,63]. It was found that, in a model of toxic demyelination in rat brain, GBP (at 300 mg/kg) significantly increased the oxidative stress developed after ethidium bromide, whereas GBP (at 100 mg/kg) did not induce oxidative stress [60]. Therefore, further studies are needed to assess the potential of GBP to reduce oxidative stress under various pathological conditions, as well as to determine the dose range to be used for neuroprotection.

Furthermore, in an era of increasing environmental awareness, it seems important that GBP may have negative consequences for living organisms [92,93]. It has been proven that the concentration of this drug currently present in the aquatic systems of developed countries can induce oxidative stress in zebrafish embryos by modifying the expression of genes responsible for maintaining adequate levels of free radicals in cells [92]. This newly discovered environmental toxicity of the drug requires close attention.

Due to ambiguous results, the question of the influence of TPM on oxidative stress requires further research [71,72,73]. However, given the mechanism of this drug based on increasing GABA-ergic neurotransmission and blocking AMPA receptors, it may be expected that TPM generally decreases oxidative stress when used in therapeutic doses.

LTG lowers oxidative stress in PTZ-induced seizures models. This effect may be related to the reduction of excitatory signaling caused by LTG [29,30]. As for OXC and FBM, studies conducted so far did not provide unequivocal results on the effects of these drugs on oxidative stress.

Two of the studies conducted so far indicate that LEV increases oxidative stress [88,89]. On the other hand, the antioxidative potential of LEV in the PTZ-induced kindling model has been reported [77]. Moreover, LEV was proven to increase GABA-ergic neurotransmission in the PTZ-induced convulsions model, which may be the potential mechanism behind its antioxidative action [83]. More studies are evidently required to further characterize the pro/antioxidative activity of this AED.

The results of this review are briefly summarized in Table 1. The conducted literature review ostensibly indicates contradictory results of studies regarding the influence of drugs increasing GABA-ergic transmission (VGB, TGB, GBP, TPM) on oxidative stress. However, it seems that the influence of GABA-ergic transmission on the production of ROS and RNS depends on the dose of the used drugs and the pathological conditions in the tissue. In the case of oxidative stress, the introduction of a drug elevating GABA concentration causes a decrease in the oxidation markers and neuroprotection [24,34,35,41,48,51,52,53,54,55,56,57,58,61]. This is evidenced by the antioxidant activity of muscimol, a GABA-A receptor agonist, and diazepam, a GABA-A positive allosteric modulator, in a mouse with immobilization stress and in PTZ-induced seizure activity, respectively [60,63]. Moreover, the GABA-A receptor antagonist, picrotoxin, abolished the antioxidant effect of GBP in the immobilization stress mouse model [52]. This means that VGB, TGB and GBP can be considered as applicable in those diseases in which oxidative stress is an important pathophysiological factor, e.g., diabetic neuropathy and retinopathy, FXS, HD, or spinal cord ischemia-reperfusion injury [24,29,30,33,35,36,41,48,51,52,53,54,55,56,57,58,61,65].

On the other hand, when a GABA-enhancing drug was applied to the healthy tissue, it tended to increase oxidative stress markers in a dose-dependent manner [13,15,35,36,61]. Elevated GABA concentration has been proven to increase the number of mitochondria and peroxisomes and can be responsible for some of the adverse effects of drugs elevating GABA-ergic neurotransmission, e.g., visual field defects caused by VGB use [14,15]. Increasing GABA-ergic transmission in epileptic regions is beneficial and may have neuroprotective effects, whereas in unaffected organs it can cause adverse effects.

**Figure 1:**
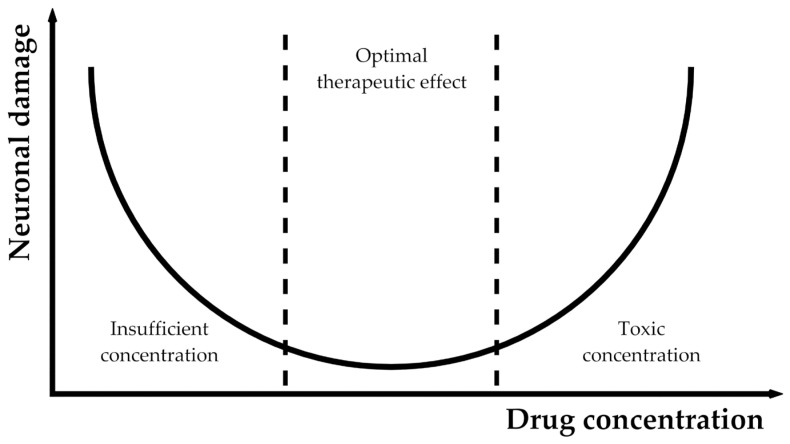
Proposed model of the “U-shaped” neuroprotection/neurodegeneration effect against oxidative stress of newer AEDs enhancing GABA-ergic neurotransmission. Based on data from [94] showing neuroprotective effects of diazepam in vitro.

It has been shown that high doses of VGB, TGB and GBP can induce oxidative stress [15,24,35,36,60,61]. Studies on diazepam have shown that it exerts a neuroprotective effect in a “U-shaped” dose-dependent manner after excitotoxic (100 μM of NMDA) or oxidative (30 μM of tertbutyl-hydroksyperoxide) stress induced in vitro in the Wistar rats’ hippocampal brain slices under constant temperature conditions (Figure 1). In this study, diazepam provided neuroprotection in the concentration range of 1–10 μM. Lower concentrations were insufficient to protect against neuronal damage, while higher concentrations were harmful [94]. Moreover, diazepam at concentrations of 25–50 μM caused enhanced apoptosis of rat cerebellar granule cells [95]. Furthermore, it was proven that diazepam provided neuroprotection following the global 5 min gerbil brain ischemia. Gerbils received two doses of 10 mg/kg i.p. of diazepam 30 and 90 min after ischemia, resulting in a more than 60% survival rate of CA1 hippocampal neurons, whereas in the control group only around 15% of neurons survived. Importantly, diazepam caused a decrease in the gerbils’ body temperature from 36.6 ± 1 °C to 33.4 ± 2 °C. In the group treated with hypothermia resembling that induced by diazepam, 42.8 ± 9.2% of live cells were observed, thus proving that diazepam-induced hypothermia is not the only neuroprotective action this drug exerts [94]. Therefore, it should be concluded that newer AEDs that enhance GABA-ergic neurotransmission may act similarly to diazepam and when used in high doses could cause neurodegeneration and, in some cases, oxidative stress.

## Figures and Tables

**Table 1:** Newer AEDs and oxidative stress.

AED	Inhibitory Transmission	Excitatory Transmission	Concentration of Oxidants under Normal Conditions (Preclinical Studies)	Concentration of Oxidants during Oxidative Stress (Preclinical Studies)	Concentration of Oxidants during Oxidative Stress (Clinical Studies)	Comments	References
VGB	↑↑↑	—	↑↑↑	↓↓↓	No data available	Animal studies have shown efficacy in FXS or in spinal cord injury. Clinical trials are needed.	[11,13,15,24]
LTG	—	↓↓↓	No data available	↓↓↓	↓↓↓ as compared to conventional AEDs	LTG effectively reduces oxidant levels, which may result in a reduction of cardiovascular risk and an increase in serum albumin concentration in patients with epilepsy.	[26,27,29,30,31]
TGB	↑↑↑	—	↑↑↑ when administered in high doses	↓↓↓	No data available	Results obtained in an animal model of HD indicate the possibility of using TGB in the treatment of HD. Clinical trials are required.	[32,33,35]
GBP	↑↑↑	↓↓↓	↑↑↑ when administered in high doses	↓↓↓	No data available	There are prospects of using GBP in treatment of HD, diabetic neuropathy, spinal cord injury.	[37,38,40,48,51,52,53,54,55,56,57,58,60,61,63]
OXC	—	—	No data available	↑	↑ in epileptic patients	Further research is needed due to conflicting data.	[7,27,71]
TPM	↑↑↑	↓↓↓	↑↑↑	↓↓↓ [72,73] ↑↑↑ [71]	No data available	Further research is needed due to conflicting data.	[27,71,72,73]
FBM	↑↑↑	↓↓↓	No data available	↓↓↓	No data available		[75,77]
LEV	↓ in healthy tissue [90] ↑ in post-epileptic tissue [91]	↓	↑	↑ [88] ↓ [77]	↑		[81,82,83,84,88,89]

↑↑↑ Increases; ↓↓↓ Decreases; ↑ Probably increased; ↓ Probably decreased; — No effect. FBM—Felbamate; GBP—Gabapentin; LTG—Lamotrigine; LEV—Levetiracetam; OXC—Oxcarbazepine; TPM—Topiramate; TGB—Tiagabine; VGB—Vigabatrin; LTG—Lamotrigine.

## Data Availability

Not applicable.
